# Meat Quality of Nellore Young Bulls—Effects of Different Days on Feed and Zilpaterol Hydrochloride Supplementation

**DOI:** 10.3390/ani11092688

**Published:** 2021-09-14

**Authors:** Mariana Caetano, Rodrigo Silva Goulart, Saulo Luz Silva, Sergio Bertelli Pflanzer, Paulo Roberto Leme, Antonio Carlos Ramos dos Santos, Dante Pazzanese Duarte Lanna

**Affiliations:** 1 Davies Livestock Research Centre, Department of Animal and Veterinary Bioscience, Roseworthy Campus, School of Animal and Veterinary Sciences, The University of Adelaide, Roseworthy 5371, Australia; mariana.caetano@adelaide.edu.au; 2 Department of Animal Science, College of Animal Science and Food Engineering, University of São Paulo, Pirassununga 13635-900, SP, Brazil; sauloluz@usp.br (S.L.S.); prleme@usp.br (P.R.L.); 3 Department of Food Technology, Faculty of Food Engineering, Campinas University, Campinas 13083-862, SP, Brazil; spflanzer@gmail.com; 4 Department of Animal Science, Luiz de Queiroz College of Agriculture, University of São Paulo, Piracicaba 13418-900, SP, Brazil; ar755@cornell.edu (A.C.R.d.S.); dplanna@usp.br (D.P.D.L.)

**Keywords:** beta-agonist, Bos indicus, fatty acid profile, meat quality, sensory panel

## Abstract

**Simple Summary:**

β-adrenergic agonists have been widely used to improve meat production efficiency and can be a valuable tool for increasing world meat supply. They act as nutrient repartitioning agents, increasing lean tissue deposition but negatively affecting meat quality, notably reducing palatability. While this metabolic modifier improves the growth performance of Zebu breeds, they can impair even more meat quality in feedlot-finished cattle. This study was developed to evaluate the impact of zilpaterol hydrochloride (ZH) feeding duration and time on feed (90 or 117 days), on meat quality, and on composition properties of meat from young Zebu bulls. Supplementing ZH resulted in a leaner and less tender and juicy meat compared to non-supplemented animals, with few differences in fatty acid composition. Feeding animals for 90 or 117 days had a small impact on meat quality attributes. Feeding ZH to Zebu bulls would require additional tenderization practices to reduce the negative effects of ZH on meat quality.

**Abstract:**

Ninety-six Nellore young bulls were fed (90 or 117 day) diets containing ZH (8.33 mg/kg) for 0, 20, 30, or 40 days to evaluate the effects of days on feed (DOF) and length of zilpaterol hydrochloride (ZH) supplementation on meat quality. At the end of feeding period, animals were slaughtered, and samples of the Longissimus muscle were collected to evaluate the chemical composition, fatty acid profile, color stability, shear force, and sensory profile. DOF did not affect chemical composition, shear force, sensory tenderness, and most of fatty acids; however, animals fed for 90 d had lower redness (*p* < 0.01), sustained juiciness (*p* < 0.01), and more flavor (*p* = 0.03) than those fed for 117 d. The ZH supplementation decreased lipid content and redness (*p* < 0.01), initial and sustained tenderness (*p* < 0.01), initial and sustained juiciness (*p* < 0.01), but increased protein (*p* < 0.01) and shear force (*p* < 0.01) as compared to non-supplemented animals. The ZH supplementation increased total PUFA, c9,c12-18:2, and 20:4-n6, and decreased c9-20:1 (*p* < 0.05). Feeding ZH impairs meat quality attributes of Nellore young bulls, regardless of duration of supplementation, while DOF has a small effect on meat quality properties.

## 1. Introduction

The Brazilian beef cattle production system is mainly based on Zebu breeds raised and finished under grazing conditions [1]. The increasing market demands for higher meat quantity and quality have also progressively increased feedlot finishing practices [2], especially using young bulls, because they grow faster than steers, utilize feed more efficiently, and have higher carcass dressing percentage and leaner meat despite poorer meat quality [3,4]. 

Growth promoters such as β-adrenergic agonists have been used to improve animal performance, feed efficiency, and lean meat yield. Zilpaterol hydrochloride (ZH) is a growth-enhancing product that belongs to the group of beta-adrenergic receptor agonists β_2_, which improves lean tissue deposition but decreases intramuscular fat content and increases shear force [5,6]. The ZH supplementation can offset high feed costs and increase profitability in cattle finished in feedlots. However, the magnitude of enhancement, the efficiency of gain, and the modification of carcass characteristics and meat quality are influenced by the duration of treatment with βAA [7].

Despite improvements in performance, ZH has been reported to decrease the sensory tenderness and juiciness scores and increase the perception of connective tissue [7,8]. This is of particular interest for Zebu (*Bos indicus*) breeds, which are extensively used in the tropics due to their adaptation to warm climates; however, they have less ability to accumulate subcutaneous and intramuscular fat, besides having a slower aging rate, which indicates meat with less sensory acceptance [9].

Previous studies have reported the effects of feeding ZH and the duration of supplementation on meat quality properties, but few of them evaluated the effect of this metabolic modifier on the meat quality of Zebu cattle [10,11,12] and non-castrated males [11,12] that have been raised and finished in tropical regions. 

Therefore, it is important to know the effects of ZH on meat quality of young Zebu bulls that have a predisposition to produce leaner and less tender meat. Thus, the objective of this study was to determine the effects of ZH feeding duration (20, 30, or 40 days) and days on the finishing diet (90 or 117 days) on the color, fatty acid profile, instrumental tenderness, and sensory attributes of the meat of young Nellore bulls.

## 2. Materials and Methods

### 2.1. Animals and Treatments

Ninety-six young Nellore bulls (377 ± 25 kg initial body weight (BW) and 24 months old) were used in a randomized complete block design with a 4 × 2 factorial arrangement to evaluate the effects of duration of ZH supplementation (0, 20, 30, or 40 days; 8.33 mg/kg; Zilmax^®^, MSD Animal Health, São Paulo, SP, Brazil) and days on feed (DOF) before slaughter (90 and 117 days). Upon arrival at the feedlot, bulls were identified with ear tags, vaccinated against clostridiosis (5 mL SC; Ourovac, Poli BT, Ourofino Saúde Animal Ltd.a., Cravinhos, SP, Brazil) and foot-and-mouth disease (5 mL SC; Bovicel, Vallée S.A., São Paulo, SP, Brazil), and dewormed with 35 g/kg ivermectin (7 mL SC; Puritec Gold, Ceva Brasil, Paulínia, SP, Brazil).

The animals were blocked by initial BW and randomly assigned to treatments. All animals were individually fed using separate pens (*n* = 48; 6 animals per treatment) and Calan gates (*n* = 48; a total of 6 bulls per treatment, with all treatments represented in each pen). 

Adaptation to a high-grain diet was carried out gradually over a 12 days period using 2 step-up diets (6 days each; Table 1). The ZH was included in the mineral premix at 8.33 mg/kg (DM basis) and mixed with all the feed ingredients before preparation of the total mixed ration. The mineral premix contained, per kilogram, 45.4 g Ca, 73.4 g S, 34.9 g P, 30.9 g Mg, 213.1 g Na, 82 mg Co, 1.871 mg Cu, 1.000 mg Fe, 130 mg I, 3.670 mg Mn, 26 mg Se, 5.564 mg Zn, 290.000 IU vitamin A, and 2.533 mg salinomycin (equivalent to 13 mg/kg of diet DM).

Diets were formulated based on the beef cattle [13] nutrient recommendations (Table 1), and feed was offered twice daily (7:00 a.m. and 4:00 p.m.) on ad libitum intake.

### 2.2. Slaughter and Sample Collection 

At the end of each DOF (*n* = 48, 90 day; *n* = 48, 117 day), finished bulls were slaughtered in a Federal Inspected Abattoir (Lins, SP, Brazil), located around 300 km from the feedlot location. After resting for about 12 h, the bulls were slaughtered following procedures established by the Sanitary and Industrial Inspection Regulations for Products of Animal Origin [14]. The animals were stunned with a captive bolt and the bleeding was performed by the severing of the carotid artery and jugular vein.

After 48 h of chilling (0–2 °C), the left strip loin from each carcass (from the 10th thoracic vertebra to the 6th lumbar vertebra) was taken, identified, individually vacuum packed, placed in a portable cooler with ice, and transported to the Meat Laboratory of the University of Campinas (Campinas, SP, Brazil).

At the laboratory, three days after slaughter, each strip loin was cut into eight steaks, from the anterior to the posterior end: four steaks (2.5 cm thick) for Warner–Bratzler shear force (WBSF) determination and cooking loss analysis; two steaks (2.5 cm thick) for sensory analysis; one steak (2.5 cm thick) for pH, chemical composition, and fatty acid profile analysis; and one steak (7 cm thick) for color stability measurement. All steaks were identified and vacuum packed. Samples used for pH, chemical composition, and fatty acid profile analysis were immediately frozen (−18 °C), and the remaining samples were aged (2 °C). Information about aging and randomization of steaks used for WBSF, sensory analysis, and color stability will be described later.

### 2.3. pH, Chemical Composition, and Fatty Acid Composition

The steaks were thawed (4 °C/24 h), and the pH was measured in duplicate by inserting a calibrated puncture pH electrode (SevenGo Duo, Mettler Toledo, Greifensee, Sweden) directly into the steak. After pH measurement, the steak was trimmed of external fat and apparent connective tissue and was ground. The lipid [15], protein, and moisture [16] contents were determined in triplicate. Fatty acids were extracted, according to Hara e Radin [17], and then methylated, according to Christie [18].

Fatty acid methyl esters were determined by gas chromatography (Finnigan Focus GC, Thermo Fisher Scientific, Waltham, CA, USA), equipped with a flame ionization detector and a 100 m CP-Sill 88 (Varian) fused silica capillary column (100 m, 0.25 mm, and 0.2 μm film thickness). 

The column oven temperature was programmed at 70 °C for 4 min, 170 °C (13 °C min^−1^) and 250 °C (35 °C min^−1^) for 5 min. The gas fluxes were 1.2 mL min^−1^ for carrier gas (He); 45 mL min^−1^ for make-up gas (N2); 40 mL min^−1^ for hydrogen, and 450 mL min^−1^ for synthetic flame gas. One µL sample was injected in split mode at 1/21. Injector and detector temperatures were 250 °C and 300 °C, respectively.

Fatty acids were identified by comparing the relative retention times of the peaks of fatty acid methyl esters with a reference compound (CRM-164, Commission of the European Communities, Community Bureau of Reference, Brussels, Belgium). Fatty acids were expressed as a percentage of overall fatty acids identified.

The total of saturated fatty acid (SFA), monounsaturated fatty acid (MUFA), and polyunsaturated fatty acids (PUFA) were obtained by the sum of individual fatty acids within each category.

Hydrogen was used as a carrier gas at a flow rate of 1.8 mL per minute. The initial temperature of the oven was 70 °C for 4 min, gradually increasing by 13 °C per minute until reaching 175 °C, and then maintained for 27 min. The temperature was then increased by 4 °C per minute until reaching 215 °C, where it was maintained for nine minutes, followed by another increase of 7 °C per minute until it reached 230 °C, where it remained for another five minutes. The temperature used for the vaporizer was 250 °C, while that for the detector was 300 °C. The identification of fatty acids was performed by comparing the retention times with fatty acid standards (Supelco TM Component FAME Mix, cat 18919 Supelco, Bellefonte, PA and Conjugated Linoleic Acid Standard, CLA, cat05632-SigmaSupelco TM Component) and those of butter (CRM-164); the percentage values of fatty acids were obtained using the Chromquest 4.1 software (Thermo Electron, Milan, Italy).

### 2.4. Color Stability

A subsample cut for color stability (7 cm thick) was aged for four more days (7 day post-mortem). Following this, samples were unpacked, and a slice (1.5 cm) was taken for color display analysis (fresh surface). The remaining part of the sample was re-packed and stored at 2 °C for an additional seven days (14 day post-mortem), after which another slice (1.5 cm tick) was taken. Each steak assigned to color stability was placed in a polystyrene tray, then wrapped with a polyvinyl chloride film and kept in a refrigerator at 4 °C, with no lights, for five days. Instrumental color was determined 30 min after display and every 24 h following the American Meat Science Association [19] protocol. The color was evaluated using a colorimeter (CM 508-d, Hunter MiniScan TMXE, Hunter Associates Laboratory, Inc., Reston, VA, USA) attached to a moisture protector accessory and previously calibrated using white and black tile standards. The color was measured in three different positions on the surface (in triplicate) by the Commission Internationale de I’Eclairage (CIE; “International Commission on Illumination”) L*, a*, and b* values, using the standard observer of 10°, illuminant D65 source, and an aperture size of 25 mm. The CIE L*, a*, and b* values were used to calculate hue angle [arctan (b*/a*)] and saturation index [(a*^2^ + b*^2^)^½^]. 

### 2.5. Warner–Bratzler and Cooking Loss

The four steaks assigned for WBSF were individually labeled, vacuum packed, and randomly allocated to aging periods of 7, 14, 21, and 28 days post-mortem at 2 °C. After reaching the desired aging time, the steaks were frozen (−18 °C) until analysis. The WBSF analysis was conducted according to the methodology described by the American Meat Science Association [19]. Twenty-four hours before the WBSF analysis, the frozen samples were transferred to a refrigerator (4 °C) and allowed to thaw. After being unpacked, the samples were weighed, and a thermocouple (model 0602.5792, Testo do Brazil, SP, Brazil) was inserted into the geometric center of the sample to monitor the internal temperature. Samples were cooked in a pre-heated (170 °C) electric oven to a temperature of 40 °C, after which samples were flipped over and cooked to a temperature of 71 °C. Samples were cooled to room temperature (22 °C), weighed, wrapped in plastic film, and cooled in a refrigerator (4 °C) for 12 h. The WBSF was determined from six replicates (1.27 cm in diameter), with the fiber direction parallel to the longest dimension of the strip and perpendicular to the direction of the blade, using a texturometer TA-XT 2i (Stable Micro Systems, Godalming, Surrey, UK) equipped with a 1 mm blade.

### 2.6. Sensory Analysis

The two steaks assigned for sensory analysis were individually labeled, vacuum packed, and randomly allocated to aging (2 °C) for 7 and 14 days post-mortem. The steaks were frozen (−18 °C) until analysis. Twenty-four hours preceding the analysis, the samples were placed in a refrigerator (4 °C) for thawing and then cooked using the same procedure described for WBSF. Cooked samples were cut into 1.5 cm × 1.5 cm × 1.5 cm pieces, avoiding fat and any visible connective tissues, and placed in glass jars with metal lids and kept in a container with a regulated thermostat (40 °C) before serving them to the panelists. Thus, eight panelists were trained in 6 sessions of 1 h. Training was performed on the evaluation of different beef muscles with differences in juiciness, tenderness, and flavor. Sensory analyses were performed in individual booths under controlled conditions of red light and temperature (22 ± 2 °C). One steak from each treatment (4 periods of ZH supplementation × 2 DOF × 2 aging times) was used in each session, with a total of 12 sessions. Samples were placed in a plastic cup with a three-digit random number for each treatment and served in random order to the panelists with filtered water and unsalted crackers to cleanse the palate between samples. The meat attributes were evaluated using the eight-point scale according to the methodology described by the American Meat Science Association [19] for initial and sustained tenderness (extremely tough—1; extremely tender–8), initial and sustained juiciness (extremely dry—1; extremely juicy—8), and beef flavor intensity (extremely weak—1; extremely strong—8).

### 2.7. Statistical Analysis

Plots of residuals and the W statistic were used to determine normality for all data, and outliers (>3 and <−3 standard deviation) were excluded from analyses.

The pH, chemical composition, and fatty acid profile data were analyzed as a randomized complete block design with a 4 × 2 factorial arrangement of treatments, considering the fixed effects of the duration of ZH supplementation (0, 20, 30, or 40 day), DOF (90 or 117 day), and ZH × DOF interaction, and the random effect of block (initial BW). The animal was the experimental unit. For traits evaluated over time (color stability, WBSF, and cooking loss), the time of measurement and 2nd and 3rd order interactions were included as fixed effects. The covariance structure of residuals was modeled, and the best fitted for every trait was used. The animal was the experimental unit. These analyses were conducted using the MIXED procedure of SAS (SAS Institute, Cary, NC, USA). 

Sensory traits were not normally distributed and were evaluated as negative binomial distribution using the GLIMMIX procedure of SAS, with ZH supplementation, DOF, and the interaction ZH × DOF as fixed effects, and the random effect of block (initial BW). 

When significant effects were observed, the means were compared by regression (days of ZH supplementation and time on display) or by Fisher’s F-test (DOF, aging period). In addition, orthogonal contrasts were used to compare: (1) control vs. ZH supplemented groups; (2) linear and quadratic effects of the ZH administration period.

## 3. Results

### 3.1. pH and Chemical Composition

No interaction was observed between the DOF and duration of ZH supplementation for pH and meat chemical composition. The pH was not affected by the DOF nor the duration of ZH supplementation (Table 2). The lipid content decreased, and protein increased linearly (*p* < 0.01) with the duration of ZH supplementation, while moisture was not affected. The DOF did not affect moisture, fat, and protein deposition.

### 3.2. Fatty Acid Composition

There was no interaction between the DOF and duration of ZH supplementation for fatty acid composition. ZH supplementation had a negligible impact on the majority of fatty acids, except for increasing the total PUFA (*p* = 0.04), c9,c12-18:2 (*p* = 0.03), and 20:4n6 (*p* = 0.05) concentrations and reducing c9-20:1 (*p* < 0.01; Table 3). 

The duration of ZH supplementation was quadratically associated with total PUFA (*p* = 0.01), c9,c12 18:2 (*p* = 0.01), 20:3n-6 (*p* < 0.01), 20:4n-6 (*p* < 0.01), and 22:5n-3 (*p* < 0.01), with higher levels for ZH-treated as compared to control animals. The concentration of c9-20:1 decreased linearly (*p* = 0.02) with the increase of ZH supplementation period.

The meat of bulls fed for 90 days had higher concentrations of 10:0 (*p* = 0.01), 18:3n-3 (*p* < 0.01), 20:5n-3 (*p* < 0.01), 22:5n-3 (*p* = 0.01), and 22:6n-3 (*p* < 0.01) than those fed for 117 days (Table 3). Even with greater concentrations of these individual fatty acids, the total amounts of PUFA, MUFA, and saturated were not affected by the DOF. Smaller concentrations of c9-14:1 (*p* < 0.01) and n-6:n-3 ratio (*p* < 0.01) were observed in young Nellore bulls fed for 90 days.

### 3.3. Color Stability 

No significant interaction among the DOF, duration of ZH supplementation, aging period, and days on display were found for meat color attributes. The b* value tended to have a quadratic association (*p* = 0.06) with the duration of ZH supplementation, decreasing from 0 to 20 days and increasing from 20 to 40 days of ZH supplementation (Table 4). The L* and a* values were linear (*p* < 0.05 and *p* < 0.01, respectively) and quadratically (*p* < 0.01) associated with the duration of ZH supplementation, with L* increasing and a* decreasing from 0 to 20 days of ZH supplementation, without changes from 20 to 40 days of ZH administration. A similar trend was observed for Chroma and Hue, which decreased from 0 to 20 days and increased from 20 to 40 days of ZH supplementation.

The meat of young bulls fed for 117 days was redder (a*; *p* < 0.01) and had lower hue values (*p* < 0.01) than those fed for 90 days, while L*, b*, and Chroma were not affected by the DOF. Meat aged for 14 days had greater L* (*p* < 0.01) and smaller a*, b*, Chroma (*p* < 0.01), and Hue values (*p* = 0.04) than those aged for 7 days. The L*, a*, Chroma, and Hue values were linear and quadratically associated with time on display (*p* < 0.01), with L* increasing from 0 to 1 day of display, with no changes until day 5; a* and Chroma increased from 0 to 1 day and decreased from 1 to 5 days, while Hue decreased from 0 to 1 day and later increased until the end of display exposure. The b* value decreased linearly, with increasing time on display (*p* < 0.01).

### 3.4. Shear Force and Cooking Loss

No significant interactions among the DOF, duration of ZH supplementation, and aging period were found for WBSF and cooking loss. 

Cooking loss was not affected by the DOF, duration of ZH supplementation, or aging period. The WBSF was quadratically associated with the duration of ZH supplementation (*p* < 0.01), with an increased WBSF from 0 to 20 days of ZH supplementation, and without changes from 20 to 40 days. ZH supplementation increased WBSF by 1.2 kg on average, compared to the non-supplemented group (*p* < 0.01), with a very low variation (0.2 kg on average) within the ZH-supplemented groups. DOF did not affect WBSF.

However, the increase of the aging period linearly reduced (*p* < 0.01) the WBSF, from 6.2 kg in non-aged samples to 4.4 kg after 28 days of aging (Table 5).

### 3.5. Sensory Panel

Initial and sustained tenderness and juiciness decreased linearly (*p* < 0.01) with an increase in the duration of ZH supplementation. However, flavor was not affected by ZH supplementation and the aging period. The DOF did not affect initial or sustained tenderness and initial juiciness, but samples of bulls fed for 117 days had higher (*p* < 0.01) sustained juiciness and beef flavor intensity (*p* = 0.03) than those fed for 90 days. Beef aged for 14 days had higher tenderness scores (initial and sustained; *p* < 0.01) than those aged for seven days, but no differences were observed for initial and sustained juiciness and flavor intensity (Table 5).

## 4. Discussion

### 4.1. Chemical Composition

Similar to previous studies [20,21,22], the intramuscular fat content decreased, and the protein content increased in ZH-supplemented bulls. However, other authors did not observe differences in lipid content according to the days of ZH supplementation, which is not in line with the results of the present study, where the lipid content decreased and the protein increased linearly as the supplementation time increased. Thus, this study confirms that ZH increases muscle deposition and consequently reduces fat in the carcass and meat; in the case of Zebu bulls, the increase in ZH supplementation time progressively favored this mechanism. Therefore, ZH supplementation for longer periods in cattle with low intramuscular fat, such as zebu breeds and bulls, may further compromise meat quality, since intramuscular fat is one of the most important traits of meat flavor. 

The effect of ZH on muscle protein deposition has been attributed mainly to the increase in the activity of calpastatin, an enzyme that inhibits the action of calpains (µ-calpain and m-calpain), decreasing protein degradation [23,24]. The change in the chemical composition of meat could also be associated with a decrease in fat deposition. Another reason for this decrease in intramuscular fat could be due to a fat-diluting effect caused by the increase of the muscle area or a decrease in the relative volume of adipocytes [6]. In this sense, the literature data are not conclusive, and it is quite possible that more than one effect affects the meat lipid content simultaneously.

On the other hand, Costa et al. [12] observed no differences in the fat deposition (subcutaneous and intramuscular), protein, and moisture content of Nellore bulls fed ZH as compared to the control. Likewise, Choi et al. [25] found no effect of ZH supplementation on the meat chemical composition of Hanwoo steers fed ZH as compared to a control group. 

Longer intensive feeding periods are associated with greater intramuscular fat depositions [26]. However, in the present study, this phenomenon was not present, even when the feeding time was increased by 27 days. The lack of difference between groups could be related to the fact that Zebu breeds and non-castrated animals have lower rates of intramuscular fat deposition as compared to castrated British cattle [27], and the additional 27 DOF was not enough to increase the intramuscular fat content.

### 4.2. Color Stability

Meat color indicates freshness and has a major impact on purchase decision [28]. Paler or darker meats as well as those with metmyoglobin content above 40% are usually rejected [29]. In the present study, the meat of ZH-supplemented bulls showed a lighter (L* value) and less red (a* value) color than the non-supplemented ones, as well as lower Chroma and Hue values, independent of display time. The lower Hue values would be indicative of lower lean discoloration for meat from ZH-supplemented animals. A prior study [30] also related lower saturation values in meat from animals that were supplemented with ZH. ZH supplementation did not affect the color stability in relation to the aging period or display exposure.

Similar to the current study, higher L* and lower a* values have been observed in the meat of ZH-supplemented animals [21]. On the other hand, other studies reported no effects of ZH supplementation on the color attributes of British steers [30] and Nellore heifers [10]. Mazon et al. [11] did not observe any effects of ZH supplementation on the L* values of the longissimus of immunocastrated or non-castrated Nellore males, while lower a* and b* values were observed for the ZH-supplemented animal. However, the same author [11] found lower Chroma for meat from animal supplemented with ZH, indicating a dull meat color, as it was found in the present study. Regarding color stability during display exposure, Rogers et al. [31] reported that the meat of animals supplemented with ZH had a greater red color stability (value of a*) when it was displayed for more than two days. However, other studies reported less color stability in the meat of animals supplemented with ZH [21,30].

The effect of ZH on muscle hypertrophy has been attributed to both the increase in the diameter of muscle fibers [4] and the increase in the incidence of fast-twitch muscle fibers [32]. The latter could explain the results found for instrumental color since the authors observed a transition from slow-twitch fibers (red fiber) to fast-twitch fibers (white fiber) with the administration of ZH. In general, the higher the incidence of fast-twitch muscle fibers, the less red the meat will be due to the lower myoglobin content [33].

In the present study, increasing the feeding time from 90 to 117 days increased the meat’s a* values and decreased the Hue values, but did not affect the L* and b* attributes. The absolute difference in the a* values was small and unlikely to have any significant effect from a commercial point of view. Similar results were reported by Sami et al. [34], who evaluated the meat quality of Simmental bulls finished for 100 or 138 days. Likewise, Keane and Allen [35] reported higher a* values in meat from animals fed for longer periods. However, some authors did not find differences in the color of feedlot-finished animals for different periods [36].

Aged meat generally has higher L*, a*, and b* values when compared to fresh, non-aged meat, and this can be explained by the greater oxygenation ability of myoglobin in packaged and vacuum-aged meats [12]. In the present study, only the color of the aged meat was evaluated, and the time of aging had a significant effect on the instrumental color, with a higher value for L* and lower values for a*, b*, Chroma, and Hue in meat aged for 14 days in comparison with meats aged for seven days. However, the numerical differences found were small, possibly due to slight difference in aging times. Franco et al. [37] did not find a significant effect on the color coordinates of the meat for different aging periods, and they attributed this response to the low oxidation rate of myoglobin in vacuum-conditioned meats. On the other hand, Mazon et al. [11] found a linear increase of Chroma and Hue when meat was aged from 0 to 21 days, which could turn the meat color from dull to bright red.

The exposure of meat to oxygen, simulating commercial conditions, had little impact on the instrumental color of the meat when it was stored for up to 5 days. These results may be associated with the lower levels of intramuscular lipids present in meat in this experiment, since higher levels of lipids could be oxidized to form free radicals, which can promote the faster accumulation of metmyoglobin due to the early reduction of metmyoglobin reducing activity [38].

### 4.3. Fatty Acid Composition

To our knowledge, few studies have evaluated the effects of ZH supplementation on the fatty acid composition of beef cattle, particularly in Bos indicus cattle. Moholisa et al. [22] compared the effect of feeding ZH to finish Bonsmara cattle in feedlots and reported higher total PUFA concentration in the *Longissimus* of ZH-treated animals as compared to those given the control diet. However, these authors did not observe any effects of the treatment on the total of SFA and MUFA. In the same work, few differences were observed for individual fatty acids; however, higher concentrations of c9,c12-18:2 were found in ZH-fed animals. These results agree with those observed in our study. On the other hand, Choi et al. [25] found no differences in any individual fatty acid or total SFA, MUFA, or PUFA in Hanwoo steers fed ZH as compared to the control group. 

According to Wood et al. [27], the overall fat content and muscle of the animal have a significant impact on the fatty acid composition because of the different fatty acid compositions of neutral lipids and phospholipids. According to these authors, the PUFA are almost exclusively restricted to the phospholipid fraction of the cell membranes, and this fraction remains fairly constant or increases little as the animals get fatter. In addition, as the animals get fatter, the neutral lipids (mainly SFA and MUFA) predominate, affecting the fatty acid composition. This fact can explain why ZH supplementation did not affect the fatty acid profile in high-fat animals [25], while in our study (leaner animals), the concentrations of c9,c12-18:2 and total PUFA were increased. Wood et al. [27] reported that in young lean animals, genetically lean animals, or animals fed a low energy diet, the lower c9-18:1 and higher 18:2n-6 content of phospholipids have a significant influence on total muscle fatty acid composition. A similar pattern observed in our study was also found by Moholisa et al. [22] in lean animals. Therefore, it is suggested that the differences in fatty acid composition could be more related to the reduction of fat deposition than to the changes in the metabolic pathways.

As the period in the feedlot increases, so does the overall body fat content of animals. Warren et al. [39] observed a significant increase of neutral lipids across breeds and age at slaughter of feedlot-finished animals, while phospholipid fractions remained constant. As a result, SFA and MUFA concentrations increased markedly, while PUFA remained constant with the increasing age at slaughter. Therefore, based on these results, it would be expected that the increase of the DOF in this study would result in increased intramuscular fat deposition, and therefore in the SFA and MUFA concentrations [40], which did not happen. The possible explanation for this could also be due to the low fat content (2.2 to 2.5%) of animals in this study, with no difference between the 90 and 117 DOF groups.

### 4.4. Shear Force

Meat tenderness is the most important characteristic noticed by consumers when evaluating meat eating quality, and it guides consumers in their acceptance and purchase decision [41]. In this sense and as established by numerous studies [4,5,20,21,30], in the current experiment, it was observed that the administration of ZH reduced the instrumental and sensorial tenderness of meat, which can negatively affect the consumers’ experience. 

In the present study, the instrumental tenderness was not dependent on the duration of ZH supplementation, diverging from the study by Rathman et al. [5], where the longer duration of ZH supplementation resulted in a linear reduction of meat tenderness, when the meat was aged by 7 and 21 days. These authors suggested that 20 days of ZH supplementation was the ideal time to minimize meat toughness problems. On the other hand, when Leheska et al. [21] were evaluating the administration of ZH in steers and heifers, they observed greater shear force when the animals were supplemented for 20 or 40 days as compared to control animals. However, these authors did not note a difference between the duration of ZH supplementation, supporting the results of the present study.

Rathman et al. [5] concluded that there is a different curve in the improvement of instrumental tenderness among animals, depending on whether they were supplemented or not with ZH. The meat of ZH-supplemented animals, even with greater shear force after slaughter, presents a more accelerated aging response, that is, a greater improvement in instrumental tenderness than that in control animals. However, even with this accelerated decline, the meat of animals supplemented with ZH remained tougher than that of control animals until 21 days of aging. These results differ from those of the present study, where no significant interaction between ZH supplementation and aging time was found. In the present trial, the meat of animals supplemented with ZH after 21 days of aging had a shear force similar to that of the meat of control animals with seven days of aging (data not shown in the table). Thus, longer aging, at least 21 days, can be a useful, low-cost, and efficient strategy to improve the tenderness of meat from animals supplemented with ZH.

### 4.5. Sensory Panel

In the present study, ZH supplementation reduced the tenderness and juiciness of meat. Similar results were reported by Cônsolo et al. [10], where meat from heifers supplemented with ZH was 37% tougher and dryer than meat from control heifers aged 0, 7, 14, or 21 days. However, the results reported in the literature with regard to the effects of ZH administration on the perception of meat sensory attributes are not completely aligned—a fact probably due to the large variations between the pre- and pos-slaughter factors evaluated in these studies such as breed and age of the animals, supplementation time, aging time, and amount of intramuscular fat. Hilton et al. [20] reported the reduced tenderness and juiciness of beef from cattle supplemented with ZH, while Garmyn et al. [42] found no difference in meat juiciness between ZH-supplemented and control animals.

The tougher meat of animals supplemented with ZH may be related to the mechanisms responsible for the increase in muscle mass. ZH increases the incidence of fast-twitch muscle fibers [32], which have a larger diameter, thus promoting an increase in muscle mass. However, fast-twitch muscle fibers have an unfavorable relationship between calpastatin and calpain for the meat aging process [43].

Numerous studies have demonstrated a decrease in myofibrillar protein fragmentation with the administration of ZH [8,24], attributed mainly to the increase in calpastatin activity [23,24], although other authors have reported that the activities of calpastatin, µ-calpain, and m-calpain do not change with the administration of ZH [20]. On the other hand, Cônsolo et al. [10] found a greater expression of calpastatin mRNA as well as µ-calpain and m-calpain, and suggested that an excessive expression of calpastatin could inhibit the activity of calpains, blocking the beneficial effects of the greater expression of calpains and their effects on the improvement of meat tenderness.

More time on feed can promote a greater deposition of subcutaneous and intramuscular fat, decreased collagen content, and increased proteolysis, measured by the myofibrillar fragmentation index [44], which would favor meat tenderness. However, in the present study, prolonging feeding for 27 additional days did not affect the instrumental tenderness, measured by WBSF or by a trained panel, which was similar to the results presented by Sami et al. [34]. On the other hand, the extended feeding period favored the juiciness and flavor of the meat, possibly due to the slightly greater deposition of intramuscular fat, even at a low amount (~2.5%) in the meat of these animals. The intramuscular fat content is considered the main factor affecting the juiciness and flavor of the meat.

## 5. Conclusions

Feeding ZH impairs meat quality attributes of non-castrated Zebu young bulls, but they are not worsened by increasing the duration of supplementation. These negative effects on meat quality were not mitigated by longer feeding periods. The DOF, as used in this study, have little impact on the meat quality attributes of leaner Zebu cattle. 

## Figures and Tables

**Table 1:** Composition of experimental diets (DM basis).

	Adaptation 1 ^1^	Adaptation 2 ^2^	Control Finishing Diet	ZH Finishing Diet
Ingredient, % of DM				
Corn silage	50.00	34.00	15.00	15.00
Finely ground dry corn	26.00	48.00	69.66	69.66
Cottonseed hulls	15.00	10.78	10.00	10.00
Soybean meal	6.00	4.00	2.00	2.00
Urea	0.70	1.00	1.20	1.20
Limestone	1.58	1.48	1.04	1.04
Potassium chloride	-	-	0.38	0.38
Sodium chloride	0.10	0.12	0.10	0.10
Dicalcium phosphate	0.10	0.10	0.10	0.10
Mineral premix ^3^	0.52	0.52	0.52	-
Mineral premix + ZH ^3,4^	-	-	-	0.52
Chemical composition, % ^5^				
DM	54.50	60.88	72.06	72.10
TDN	76.07	79.09	82.86	82.86
Crude protein	14.44	14.43	14.49	14.30
Ether extract	4.56	4.14	5.58	5.14
NDF	34.86	27.18	22.09	21.79
ADF	20.50	17.19	11.23	11.21

^1^ Diet was offered to the first 6 day of adaptation; ^2^ Diet was offered from 7 until 12 day; ^3^ Mineral premix contained, per kilogram, 45.4 g Ca, 73.4 g S, 34.9 g P, 30.9 g Mg, 213.1 g Na, 82 mg Co, 1.871 mg Cu, 1.000 mg Fe, 130 mg I, 3.670 mg Mn, 26 mg Se, 5.564 mg Zn, 290.000 IU vitamin A, and 2.533 mg salinomycin (equivalent to 13 mg/kg of diet DM); ^4^ The zilpaterol hydrochloride (ZH) premix supplied ZH at 8.33 mg/kg (DM basis); ^5^ Dry matter (DM), Neutral detergent fiber (NDF), Acid detergent fiber (ADF),Total digestible nutrients (TDN) were calculated according to [13].

**Table 2:** Least square means, standard error of the mean (SEM), and probabilities (Pr > F) of pH and chemical composition of meat samples according to the duration of zilpaterol hydrochloride (ZH) supplementation and days on feed.

Traits	pH	Lipid, %	Protein, %	Moisture, %
Duration of ZH Supplementation, Days				
0	5.6	2.7	22.0	73.9
20	5.5	2.4	22.3	74.0
30	5.5	2.1	22.4	74.1
40	5.5	2.0	22.6	74.1
SEM	0.02	0.13	0.17	0.18
Pr > F linear	0.72	<0.01	0.02	0.46
Pr > F quadratic	0.96	0.45	0.17	0.63
Control vs. ZH ^a^	0.68	<0.01	<0.01	0.40
Days on feed				
90	5.6	2.2	22.4	74.1
117	5.5	2.5	22.2	74.0
SEM	0.01	0.09	0.12	0.14
Pr > F	0.41	0.08	0.08	0.55

^a^ Probability of orthogonal contrast: control vs. ZH-supplemented bulls.

**Table 3:** Least square means, standard error of the mean (SEM), and probabilities (Pr > F) of fatty acid composition (%) of meat samples according to the duration of zilpaterol hydrochloride (ZH) supplementation and days on feed.

Fatty Acids, %	Days on Feed		SEM	Pr > F	Duration of ZH Supplementation, Days				SEM	Pr > F		
	90	117			0	20	30	40		0 vs. ZH ^1^	L ^2^	Q ^3^
Saturated	47.6	46.8	0.81	0.50	47.6	46.9	47.0	47.3	1.15	0.68	0.87	0.66
10:0	0.18	0.10	0.03	0.01	0.12	0.14	0.17	0.14	0.04	0.44	0.62	0.42
12:0	0.16	0.12	0.02	0.14	0.12	0.14	0.16	0.14	0.02	0.30	0.34	0.71
14:0	3.35	3.50	0.10	0.30	3.33	3.57	3.40	3.41	0.14	0.40	0.92	0.40
15:0 iso	0.08	0.08	0.01	0.99	0.07	0.08	0.07	0.09	0.01	0.65	0.30	0.18
15:0 anteiso	0.10	0.10	0.01	0.57	0.09	0.11	0.09	0.11	0.01	0.33	0.31	0.75
16:0	25.9	25.7	0.53	0.80	26.4	25.7	25.5	25.6	0.75	0.34	0.42	0.60
17:0	1.26	1.18	0.05	0.09	1.28	1.19	1.20	1.21	0.06	0.15	0.38	0.26
18:0	15.6	15.1	0.36	0.32	15.3	15.0	15.5	15.7	0.50	0.83	0.47	0.65
Monounsaturated	42.5	43.6	0.72	0.29	43.8	42.5	42.6	43.4	1.01	0.41	0.84	0.29
c9-14:1	0.65	0.77	0.03	<0.01	0.70	0.76	0.67	0.71	0.04	0.66	0.81	0.72
c9-16:1	2.69	2.87	0.07	0.07	2.74	2.87	2.72	2.78	0.10	0.68	0.92	0.71
c9-17:1	0.80	0.76	0.04	0.27	0.79	0.78	0.77	0.77	0.05	0.73	0.69	0.92
t6,t7,t8,t9-18:1	0.32	0.34	0.02	0.52	0.32	0.28	0.31	0.40	0.03	0.69	0.10	0.02
t10,t11,t12-18:1	1.61	1.53	0.12	0.63	1.64	1.39	1.34	1.89	0.16	0.59	0.58	0.02
c9-18:1	34.5	35.4	0.68	0.30	35.7	34.4	34.6	34.9	0.91	0.30	0.61	0.37
c11-18:1	0.92	0.86	0.05	0.35	0.85	0.90	0.95	0.87	0.07	0.42	0.58	0.41
c9-20:1	0.16	0.17	0.01	0.25	0.19	0.16	0.15	0.15	0.01	<0.01	0.02	0.08
Polyunsaturated	9.84	9.56	0.44	0.66	8.59	10.6	10.4	9.25	0.62	0.04	0.52	0.01
c9,c12-18:2	6.95	7.06	0.32	0.80	6.22	7.55	7.53	6.71	0.44	0.03	0.45	0.01
c9,t11-18:2	0.22	0.21	0.02	0.52	0.21	0.21	0.20	0.24	0.04	0.59	0.21	0.28
18:3n-3	0.20	0.13	0.01	<0.01	0.16	0.18	0.17	0.17	0.01	0.32	0.49	0.57
20:3n-6	0.23	0.20	0.02	0.27	0.18	0.26	0.24	0.20	0.03	0.11	0.85	<0.01
20:4n-6	1.27	1.23	0.08	0.72	1.06	1.42	1.38	1.13	0.11	0.05	0.74	<0.01
20:5n-3	0.21	0.13	0.01	<0.01	0.16	0.20	0.16	0.15	0.02	0.63	0.46	0.24
22:5n-3	0.67	0.54	0.01	0.01	0.53	0.67	0.65	0.57	0.05	0.09	0.63	0.04
22:6n-3	0.07	0.04	0.01	<0.01	0.04	0.07	0.06	0.05	0.01	0.12	0.71	0.07
Unsaturated:saturated	1.12	1.16	0.03	0.35	1.14	1.14	1.14	1.13	0.04	0.88	0.82	0.92
n-6:n-3	7.69	10.20	0.33	<0.01	8.78	8.94	9.33	8.78	0.58	0.29	0.37	0.10

^1^ Probability of orthogonal contrast: control vs. ZH-supplemented bulls. ^2^ Linear effect of the days of ZH supplementation; ^3^ Quadratic effect of the days of ZH supplementation.

**Table 4:** Least square means, standard error of the mean (SEM), and probabilities (Pr > F) of color attributes according to the duration of zilpaterol hydrochloride (ZH) supplementation, days on feed, aging period, and days on display.

Duration of ZH Supplementation, Days	L*	a*	b*	Chroma	Hue
0	40.2	20.4	16.8	25.4	39.1
20	41.3	19.2	16.4	23.2	38.6
30	40.8	19.8	16.5	24.7	39.1
40	40.8	19.6	16.7	24.8	39.9
SEM	0.35	0.19	0.23	0.13	0.09
Pr > F linear	0.02	<0.01	0.96	<0.02	<0.01
Pr > F quadratic	<0.01	<0.01	0.06	<0.01	<0.01
Pr > F Control vs. ZH ^a^	<0.01	<0.01	0.09	<0.01	0.03
Days on feed					
90	40.8	19.4	16.6	24.6	40.1
117	40.8	20.2	16.7	24.5	38.2
SEM	0.34	0.17	0.22	0.09	0.06
Pr > F	0.67	<0.01	0.76	0.51	<0.01
Aging period, days					
7	40.5	20.6	17.3	25.4	39.3
14	41.0	19.0	16.0	23.7	39.1
SEM	0.34	0.16	0.21	0.09	0.06
Pr > F	<0.01	<0.01	<0.01	<0.01	0.04
Time on display, days					
0	40.0	20.4	17.1	24.8	38.9
1	40.8	21.3	17.0	25.5	37.9
2	41.0	20.3	16.6	24.8	38.4
3	41.0	19.6	16.5	24.5	39.4
4	40.9	18.9	16.3	24.0	39.8
5	40.9	18.2	16.2	23.6	40.5
SEM	0.36	0.21	0.24	0.15	0.11
Pr > F Linear	<0.01	<0.01	<0.01	<0.01	<0.01
Pr > F Quadratic	<0.01	<0.01	0.77	<0.01	<0.01

^a^ Probability of orthogonal contrast: control vs. ZH-supplemented bulls.

**Table 5:** Least square means, standard error of the mean (SEM), and probabilities of the Warner–Bratzler shear force (WBSF), cooking loss, and sensory attributes of meat samples according to the duration of zilpaterol hydrochloride (ZH) supplementation, days on feed, aging period, and days on display.

	WBSF, kg	Cooking Loss, %	Sensory Traits				
			Initial Tenderness ^b^	Sustained Tenderness ^b^	Initial Juiciness ^b^	Sustained Juiciness ^b^	Flavor ^c^
Duration of ZH Supplementation, Days							
0	4.3	19.0	6.3	6.0	5.9	5.9	5.8
20	5.6	19.2	5.7	5.5	5.7	5.5	5.8
30	5.4	19.5	5.7	5.4	5.5	5.4	5.8
40	5.6	19.0	5.2	4.8	5.4	5.4	5.8
SEM	0.20	0.51	0.15	0.14	0.10	0.09	0.07
Pr > F Linear	<0.01	0.87	<0.01	<0.01	<0.01	< 0.01	0.84
Pr > F Quadratic	<0.01	0.30	0.64	0.93	0.99	0.12	0.87
Pr > F Control vs. ZH ^a^	<0.01	0.55	<0.01	<0.01	<0.01	<0.01	0.92
Days on feed							
90	5.2	19.3	5.61	5.35	5.67	5.41	5.70
117	5.3	19.0	5.89	5.51	5.60	5.65	5.86
SEM	0.17	0.44	0.11	0.09	0.070	0.069	0.053
Pr > F	0.58	0.43	0.07	0.28	0.48	<0.01	0.03
Aging period, days							
7	6.2	19.8	5.60	5.27	5.61	5.46	5.78
14	5.5	18.7	5.90	5.60	5.65	5.60	5.78
21	4.9	18.7					
28	4.4	19.5					
SEM	0.20	0.51	0.09	0.08	0.067	0.070	0.049
Pr > F Linear	<0.01	0.43					
Pr > F Quadratic	0.56	0.08					
Pr > F			<0.01	<0.01	0.73	0.10	0.93

^a^ Probability of orthogonal contrast: control vs. ZH-supplemented bulls. ^b^ Eight-point structured scale ranging from (1) extremely tough or dry to (8) extremely tender or juicy; ^c^ Eight-point structured scale ranging from (1) extremely weak to (8) extremely strong.

## Data Availability

The data presented in this study are available on request from the corresponding author.
